# Linking biomimetic binding measurements to pharmacokinetic models of volume of distribution and hepatic clearance

**DOI:** 10.5599/admet.3311

**Published:** 2026-04-29

**Authors:** Klara Valko

**Affiliations:** Bio-Mimetic Chromatography, Ltd. Business &Technology Centre, Bessemer Drive, Stevenage, Herts. SG1 2DX United Kingdom

**Keywords:** Non-specific binding, pharmacokinetics, tissue binding, plasma protein binding, biomimetic chromatography

## Abstract

**Background and purpose:**

The steady-state volume of distribution reflects the extent to which drugs partition between plasma and tissues and is closely related to the tissue-to-plasma partition coefficient. In pharmacokinetics, different modelling frameworks have led to ongoing debate regarding the role of plasma protein binding and the importance of unbound drug exposure in determining both distribution and clearance. This review aims to clarify the relationship between distribution and elimination models and to assess how experimental binding measurements can support their mechanistic interpretation.

**Experimental approach:**

Established pharmacokinetic relationships linking volume of distribution (*V*_d_), clearance and half-life were analysed alongside biomimetic chromatographic measurements of drug binding to human serum albumin and phospholipid membranes using immobilized artificial membrane (IAM) chromatography. Literature data for marketed drugs were evaluated to examine how these experimental descriptors relate to distribution and clearance behaviour.

**Key results:**

The analysis shows that distribution and clearance models describe complementary aspects of drug disposition rather than contradictory processes. Tissue binding, as reflected by phospholipid affinity measured by IAM chromatography, plays a dominant role in determining *V*_d_, while plasma protein binding influences both distribution and clearance through its effect on the fraction of drug unbound in plasma. The apparent paradox of plasma protein binding arises because changes in unbound fraction affect both processes simultaneously.

**Conclusion:**

This work demonstrates that biomimetic binding measurements provide a mechanistically meaningful bridge between physicochemical properties and pharmacokinetic behaviour. By integrating experimental binding data with pharmacokinetic models, the study advances understanding of how distribution and clearance are linked, supporting more informed decision-making in early drug discovery while highlighting that clearance remains influenced by additional factors beyond non-specific binding.

## Introduction

**Figure 1.**
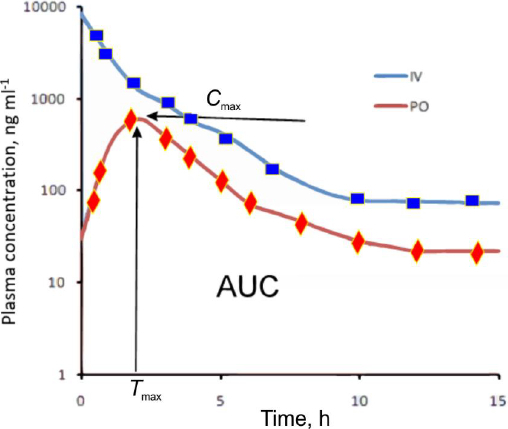
Hypothetical plasma concentration - time profile after intravenous (IV) and oral (PO) administration. *T*_max_ is the time after administration when the plasma concentration reaches the maximum (*C*_max_)

The basis of pharmacokinetic analysis of drug disposition is the plasma concentration-time profile following drug administration. From this curve, key pharmacokinetic parameters such as the area under the curve (AUC), clearance, half-life, and volume of distribution can be determined (Figure 1). The concentration-time curve is governed primarily by drug absorption and drug elimination. Following intravenous administration, distribution between plasma and tissues, as well as elimination processes, dominate the concentration-time profile. At the time of maximum concentration (*C*_max_), the rate of drug input equals the rate of drug elimination [1,2].

The apparent volume of distribution (*V*_d_) relates the amount of drug in the body to the measured plasma concentration and, for an intravenous dose, can be approximated by Equation (1).



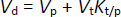

(1)


where *V*_p_ is the plasma volume, *V*_t_ is the tissue volume, and *K*_t/p_ is the tissue-to-plasma partition coefficient. This formulation shows that the apparent volume of distribution is proportional to the extent of drug partitioning into tissues relative to plasma.

The volume of distribution (*V*_d_) in which the drug would need to be uniformly distributed to produce the observed plasma concentration can be defined by Equation (2):



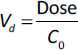

(1)


where *C*_0_ is the initial plasma concentration. The Dose is the amount of the administered drug. A mechanistic interpretation of the volume of distribution relates this parameter to the partitioning of the drug between plasma and tissue compartments.

Drug distribution is largely governed by non-specific binding interactions with biological macromolecules. In plasma, binding occurs primarily to serum albumin and α1-acid glycoprotein, whereas in tissues, drugs interact extensively with phospholipid membranes and other cellular components. These interactions determine the fraction of drug unbound in plasma (*f*_u_*)* and the fraction unbound in tissues (*f*_u,t_). Consequently, the volume of distribution can be expressed conceptually [3] as described by Equation (3).



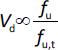

(3)


Compounds with low values of *V*_d_ remain largely confined to the plasma compartment, whereas compounds with high values of *V*_d_ partition extensively into tissues.

Drug binding processes also influence drug elimination. In the well-stirred model of hepatic clearance [4], the fraction of drug unbound in plasma directly affects the rate at which the drug is available for metabolism and elimination, as described by Equation (4).



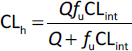

(4)


where *Q* is hepatic blood flow and CL_int_ is the intrinsic metabolic clearance. Thus, plasma protein binding affects both distribution and clearance, linking these pharmacokinetic processes through the underlying binding equilibria.

**Figure 2.**
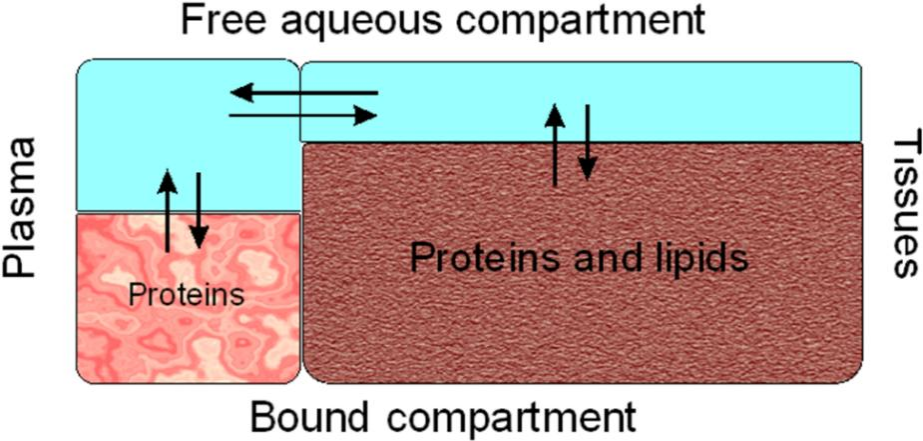
The drug molecule distributes between the tissue and the plasma compartment, which is proportional to the steady-state volume of distribution (*V*_ss_), while the distribution between the unbound compartment (free aqueous compartment) and the bound compartment in the plasma and the tissue together is proportional to the unbound volume of distribution

The unbound volume of distribution (*V*_du_) is defined by Equation (5), where *C*_free_ is the free (unbound) plasma concentration of the drug in the plasma.



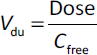

(5)


and reflects the distribution of the pharmacologically active free drug. According to the free drug hypothesis [5], the unbound drug concentration at the site of action determines receptor binding and pharmacological activity. Figure 2 shows the equilibrium process for a drug molecule.

However, increasing the unbound fraction in plasma may simultaneously increase drug clearance in the well-stirred model, creating an apparent contradiction regarding whether plasma protein binding should be minimized or optimized during drug design.

In this review, we examine the relationship between distribution models and clearance models and show that these approaches do not contradict each other but rather describe different aspects of drug disposition. We further discuss how experimental measurements of albumin and phospholipid binding using biomimetic chromatography provide mechanistic insight into the determinants of both tissue distribution and plasma protein binding. These measurements can therefore support the design of drug molecules with improved pharmacokinetic efficiency, enabling lower doses while maintaining adequate drug concentrations at the site of action.

**Figure 3.**
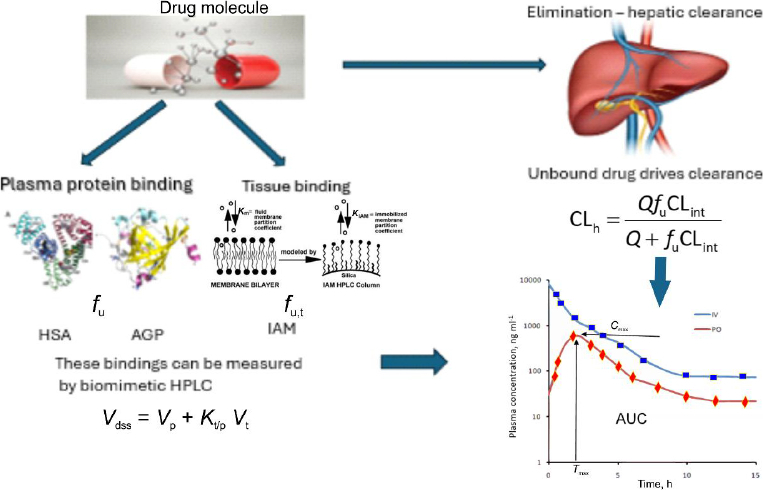
Drug binding to plasma proteins and tissue phospholipids determines the fraction of drug unbound in plasma (*f*_u_) and tissues (*f*_u,t_). These interactions govern tissue partitioning and, therefore, the steady-state volume of distribution (*V*_d_). The unbound fraction in plasma also controls hepatic clearance through the well-stirred model. Biomimetic chromatography measurements using albumin and phospholipid stationary phases provide experimental descriptors linking binding interactions to pharmacokinetic parameters. HSA - human serum albumin; AGP - α1-acid glycoprotein, IAM - immobilized artificial membrane

Figure 3 depicts the relationships between these pharmacokinetic models that will be discussed in this paper.

## Discussions

The pharmacokinetic behaviour of a drug is governed by two fundamental and largely independent parameters: the volume of distribution (*V*_d_) and systemic clearance (CL). *V*_d_ and clearance are conceptually independent parameters, although both are influenced by the fraction of drug unbound and may therefore exhibit indirect relationships. The volume of distribution reflects the extent of drug partitioning between plasma and tissues and therefore determines the relationship between administered dose and the initial plasma concentration.

In contrast, clearance describes the efficiency of drug elimination processes and governs the rate at which a drug is removed from the systemic circulation. These two parameters play distinct roles in dose selection: *V*_d_ primarily influences the loading dose, required to achieve a target plasma concentration, while clearance determines the maintenance dose and dosing frequency necessary to sustain that concentration at steady state. The interplay between *V*_d_ and CL defines the elimination half-life (*t*_1/2_), as described by the relationship in Equation (6) linking distribution and elimination processes.



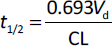

(6)


**Figure 4.**
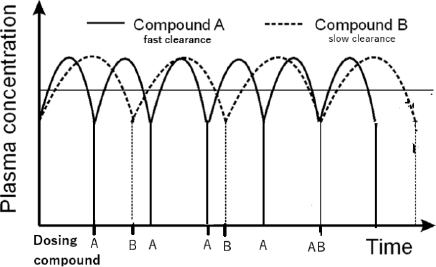
The plasma concentration - time profile for a hypothetical drug with fast (compounds A) and slow clearance (compound B)

While drug discovery strategies often emphasize clearance as the dominant determinant of dose, particularly in medicinal chemistry optimization, non-specific binding and distribution processes, as reflected in *V*_d_, also critically influence drug exposure and free drug concentrations *in vivo*. Therefore, a mechanistic understanding of both parameters is essential for rational dose prediction and compound optimization. Figure 4 shows the effect of clearance on the dosing regimen.

Table 1 shows a comparison of the roles of volume of distribution and clearance in dose estimation. Both parameters are important to know in early drug discovery. Systemic clearance is inherently more complex to predict, as elimination pathways may involve hepatic metabolism, renal excretion, or both, and these processes often vary between compounds. In contrast, the volume of distribution and its underlying determinants, tissue binding and plasma protein binding, can be experimentally estimated using biomimetic chromatographic measurements [7,8].

**Table 1.** The role of the volume of distribution (*V*_d_) and systemic clearance (CL) in dose estimation. LD is the loading dose, MD is the maintenance dose, *C* stands for the plasma concentration, *F* is the bioavailability, *C*_ss_ is the steady state plasma concentration, τ is the dosing interval in hour

	Volume of distribution	Systemic clearance
Primary use	Calculating LD	Calculating MD
Formula	LD = *V*_d_*C*_target_	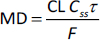
Focus	How strong is the tissue distribution	How fast the drug is removed from the body (rate)
Clinical impact	How much drug is needed to fill the body	How much drug is needed to maintain drug performance
Key factors	Tissue binding	Clearing organ function, liver, kidney

The relationship between distribution and clearance is further mediated by the fraction of drug unbound in plasma (*f*_u_), which represents the pharmacologically active and metabolically available fraction. According to the well-stirred model, hepatic clearance is proportional to the product of *f*_u_ and intrinsic clearance, indicating that only the unbound drug is available for elimination. Consequently, non-specific binding processes that reduce the free fraction of the drug may also reduce apparent clearance. This suggests that biomimetic measurements of albumin and phospholipid binding, which together determine the overall extent of drug binding in plasma and tissues, may provide indirect insight into clearance behaviour. While intrinsic clearance remains governed by enzymatic activity, the availability of free drug for elimination is modulated by binding equilibria, linking distribution and clearance through a common mechanistic framework.

Plasma protein binding also presents an apparent paradox in pharmacokinetics. Increasing the fraction of drug unbound in plasma increases tissue distribution, leading to a higher steady-state volume of distribution. At the same time, as described in the well-stirred model of hepatic clearance [4], the unbound fraction directly determines the rate at which a drug is available for metabolism, resulting in increased clearance. Consequently, decreasing plasma protein binding may simultaneously increase both distribution and elimination, resulting in a modest net effect on systemic exposure. This apparent contradiction has led to debate regarding the importance of plasma protein binding in drug discovery and whether highly bound compounds should be avoided or optimized.

A key aspect often overlooked in this discussion is that drug distribution is not governed solely by plasma protein binding but also by non-specific interactions with tissue components, particularly phospholipid membranes.

**Figure 5.**
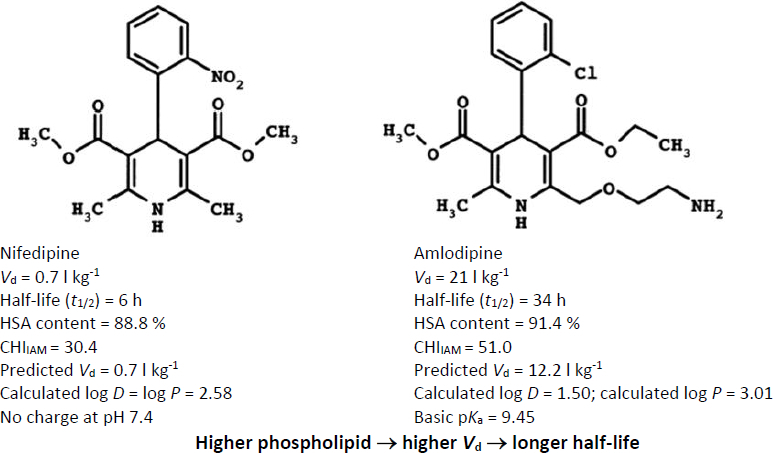
An example illustrating the influence of phospholipid binding on drug distribution. Structural modifications between nifedipine and amlodipine lead to a substantial increase in phospholipid membrane affinity as measured by IAM chromatography. While plasma protein binding remains similar for the two compounds, the increased phospholipid interaction leads to a marked increase in the steady-state volume of distribution and the elimination half-life. This example illustrates the dominant role of tissue binding in determining drug distribution and highlights the value of biomimetic chromatography measurements for predicting pharmacokinetic properties

The importance of tissue binding in determining drug distribution can be illustrated by the comparison between nifedipine and amlodipine (Figure 5).

Although both compounds show very similar plasma protein binding (~90 % bound to albumin), their pharmacokinetic behaviour differs dramatically. Nifedipine has a relatively small volume of distribution (0.7 l kg^-1^) and a half-life of approximately 6 hours, whereas amlodipine exhibits an extremely large volume of distribution (21 l kg^-1^) and a half-life of approximately 34 hours. Data were taken from reference [9]. The key difference between the two molecules is their interaction with phospholipid membranes, which is reflected in the much higher immobilized artificial membrane (IAM) chromatographic index for amlodipine. This increased phosphor-lipid affinity promotes extensive tissue partitioning, leading to a large increase in volume of distribution and prolonged half-life. These observations demonstrate that tissue binding rather than plasma protein binding is often the dominant determinant of drug distribution.

The literature values of *V*_d_, total CL, half-life (*t*_1/2_) and measured HAS and IAM binding data of a set of marketed drugs were analysed to support the above discussion. The data are compiled in Table 2.

**Table 2.** The volume of distribution (*V*_d_), total clearance (CL), half-life (*t*_1/2_), albumin binding (log *K*_HSA_) and phospholipid binding (chromatographic hydrophobicity index - CHI_IAM_) values for a diverse set of marketed drugs. (Data obtained from DrugBank.com, Bamethan’s clearance data is obtained from dog, The log *K*_HSA_ and CHI_IAM_ data were obtained from [10]) Compounds are classified as acidic, basic, neutral, or weakly ionisable based on their predominant ionisation state at physiological pH 7.4, considering their lipophilicity measured at low, neutral and high pHs

Drug name	CAS NO	*V*_d_ / l kg^-1^	CL / (ml min^-1^) kg^-1^	Half-life, h	log *K*_HSA_	CHI_IAM_	Acid/base
Acecainide	32795-44-1	1.5	2.68	8.06	0.52	23.3	Basic
Acetanilide	103-84-4	0.7	0.30	3.19	0.43	10.7	Neutral
Acetazolamide	59-66-5	0.2	1.10	6.00	1.44	1.66	Neutral
Alclofenac	22131-79-9	0.1	8.88	2.87	5.40	17.8	Acidic
Amoxapine	14028-44-5	16.0	16.6	9.00	2.70	56.5	Basic
Aspirin	50-78-2	0.2	12.00	0.25	3.33	-1.7	Acidic
Bamethan	3703-79-5	3.7	4.60	2.50	0.42	18.6	Basic
Betamethasone	378-44-9	1.4	3.00	12.47	1.39	31.7	Neutral
Carbamazepine	298-46-4	1.4	0.40	38.00	1.81	26.5	Neutral
Cefixime	79350-37-1	0.3	4.74	3.50	1.23	-3.8	Acidic
Chlorpromazine	50-53-3	21	12.30	29.17	3.87	61.9	Basic
Cinoxacin	28657-80-9	0.3	2.10	1.50	1.27	1.6	Acidic
Colchicine	64-86-8	1.4	12.10	28.81	0.91	23.7	Neutral
Cytarabine	147-94-4	2.5	38.00	0.17	0.24	-15.0	Weak base
Diazoxide	364-98-7	0.2	0.06	28.00	1.87	24.7	Weak acid
Diclofenac	15307-86-5	0.2	3.81	2.00	5.58	33.5	Acidic
Diflunisal	22494-42-4	0.1	0.15	10.00	5.18	32.5	Acidic
Diprophylline	479-18-5	0.8	4.76	1.80	0.24	-4.0	Neutral
Ethinyl estradiol	57-63-6	3.0	3.92	8.40	4.24	46.8	Neutral
Famotidine	76824-35-6	1.3	5.00	3.00	0.50	15.7	Weak base
Felodipine	72509-76-3	10.0	11.43	23.48	4.15	46.1	Neutral
Fenoprofen	31879-05-7	0.1	0.60	3.00	6.11	17.2	Acidic
Finasteride	98319-26-7	1.1	2.36	7.00	2.32	38.9	Neutral
Floxacillin	5250-39-5	0.2	1.91	1.00	3.55	23.4	Acidic
Flumazenil	78755-81-4	1.0	12.8	1.00	0.65	18.4	Neutral
Furosemide	54-31-9	0.2	1.75	1.50	4.01	21.4	Acidic
Gemfibrozil	25812-30-0	0.1	0.36	1.40	8.50	32.9	Acidic
Glipizide	29094-61-9	0.2	0.71	3.50	4.05	21.1	Acidic
Hydrochlorothiazide	58-93-5	3.0	4.05	5.60	0.8	15.9	Weak acid
Hydrocortisone	50-23-7	0.4	4.33	1.90	1.05	27.9	Neutral
Imipramine	50-49-7	21.0	16.7	12.00	2.59	51.6	Basic
Indomethacin	53-86-1	0.2	0.38	4.50	5.59	25.3	Acidic
Isradipine	75695-93-1	4.0	20.00	1.70	3.36	40.0	Neutral
Ketoconazole	65277-42-1	2.4	2.06	8.00	3.3	42.9	Weak base
Ketoprofen	22071-15-4	0.1	1.33	2.60	5.40	21.9	Acidic
Labetalol	36894-69-6	7.0	21.40	3.22	1.80	43.9	Amphoteric
Lignocaine	137-58-6	1.5	15.00	1.75	0.52	32.6	Basic
Methylprednisolone	83-43-2	0.7	5.60	2.30	1.53	32.1	Neutral
Metronidazole	443-48-1	0.8	1.53	7.30	0.28	-3.3	Weak base
Minoxidil	38304-91-5	3.0	5.03	4.20	0.72	19.0	Weak base
Nabumetone	42924-53-8	0.8	0.35	24.00	3.41	38.4	Neutral
Nadolol	42200-33-9	2.0	3.28	22.00	2.14	20.2	Basic
Nicardipine	55985-32-5	1.7	6.60	8.60	3.55	45.9	Weak base
Nifedipine	21829-25-4	1.0	8.21	2.00	1.95	29.0	Neutral
Nitrendipine	39562-70-4	3.8	18.70	19.00	3.37	40.5	Neutral
Papaverine	58-74-2	1.5	11.94	1.50	2.70	34.4	Weak base
Pentoxifylline	6493-05-6.	2.4	0.95	0.60	0.39	12.0	Neutral
Perphenazine	58-39-9	22.5	23.80	9.50	4.03	56.3	Basic
Phenytoin	57-41-0	0.7	0.70	23.00	2.02	31.6	Weak acid
Pindolol	13523-86-9	1.5	9.00	3.50	0.63	42.0	Basic
Prazosin	19216-56-9	0.5	3.09	2.50	2.40	31.6	Weak base
Prednisolone	50-24-8	1.6	2.14	3.00	0.95	28.0	Neutral
Prednisone	53-03-2	0.9	0.03	3.50	1.08	25.9	Neutral
Primidone	125-33-7	0.6	0.80	12.50	0.42	8.9	Neutral
Probenecid	57-66-9	0.2	12.50	8.00	4.01	20.1	Acidic
Procainamide	614-39-1	2.2	6.67	4.00	0.49	19.9	Basic
Propanolol	525-66-6	4.0	17.5	4.00	1.67	50.8	Basic
Propylthiouracil	51-52-5	0.4	1.73	1.50	1.02	3.9	Weak acid
Proxyphylline	603-00-9	0.6	1.17	1.50	3.21	1.1	Neutral
Sulfachlorpyridazine	80-32-0	0.1	2.00	5.74	3.02	6.0	Acidic
Sulfameter	651-06-9	0.3	0.165	5.81	2.20	3.7	Acidic
Sulfamethoxypyridazine	80-35-3	0.2	0.73	45.00	2.44	9.6	Weak acid
Sulfinpyrazone	57-96-5	0.1	0.43	5.50	4.27	26.1	Acidic
Sulfisoxazole	127-69-5	0.4	0.01	5.50	2.66	2.9	Acidic
Sulphadimethoxine	122-11-2	0.2	0.33	30.00	3.68	10.8	Acidic
Sulpiride	15676-16-1	2.5	1.80	7.50	0.71	25.5	Basic
Tamoxifen	10540-29-1	55.0	2.70	144.00	4.82	58.7	Basic
Theobromine	83-67-0	0.8	1.39	8.00	0.27	-4.1	Weak acid
Tolfenamic Acid	13710-19-5	0.2	2.21	3.50	6.60	36.6	Acidic
Trazodone	19794-93-5	1.0	1.26	7.50	3.06	36.3	Weak base
Trimethoprim	738-70-5	1.3	1.75	9.00	0.95	20.8	Weak base
Warfarin	81-81-2	0.2	0.25	40.00	4.16	19.9	Acidic
Zolmitriptan	139264-17-8	7.0	25.90	3.00	0.76	33.7	Basic

**Figure 6.**
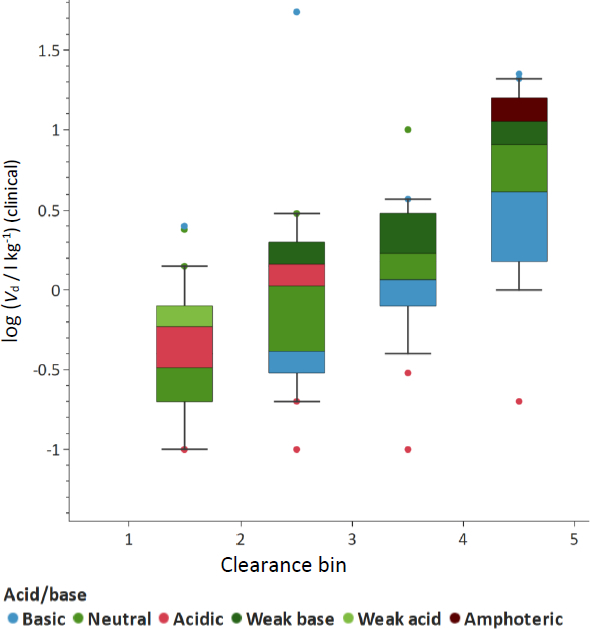
The trend between total clearance and the clinical steady-state volume of distribution. We have used four bins based on quartiles based on the data in Table 2.

**Figure 7.**
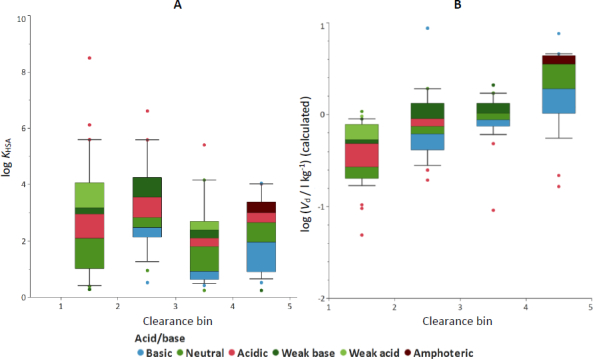
A - the trend between the binned total clearance and the albumin binding; B - the difference between the IAM binding representing the tissue partition that provided an estimated volume of distribution (log *V*_d_). The estimated and clinical log *V*_d_ values showed a good correlation as it was published earlier. bin 1: CL ≤ 0.95 (ml min^-1^) kg^-1^, bin 2: 0.95 < CL ≤ 2.68 (ml min^-1^) kg^-1^, bin 3: 2.68 < CL ≤ 8.88 (ml min^-1^) kg^-1^, bin 4: CL > 8.88 (ml min^-1^) kg^-1^

Analysis of the compiled dataset reveals that systemic clearance is associated with both the volume of distribution and biomimetic binding descriptors. A positive relationship between *V*_d_ and clearance was observed (Figure 6), consistent with the influence of the fraction of drug unbound on both distribution and elimination processes. For graphical comparison, total clearance values were divided into four approximately equally populated bins using quartile-based cut-offs. The resulting clearance categories were bin 1 is CL ≤ 0.95 (ml min^-1^) kg^-1^; bin 2, 0.95 < CL ≤ 2.68 (ml min^-1^) kg^-1^; bin 3, 2.68 < CL ≤ 8.88 (ml min^-1^) kg^-1^ and bin 4, CL > 8.88 (ml min^-1^) kg^-1^. This binning was used only for visualization and trend assessment, not as a mechanistic classification of clearance. While albumin binding alone showed limited correlation with clearance (Figure 7a), the difference between the phospholipid binding measured by IAM chromatography and the albumin binding (log *K*_HSA_) that provided the calculated log *V*_ss_ demonstrated a more pronounced relationship, particularly for basic compounds (Figure 7b).

So, increasing the fraction of drug unbound in plasma tends to increase both tissue distribution and clearance. This means that reducing plasma protein binding does not necessarily improve systemic exposure, because any increase in free concentration may be offset by faster elimination. A key reason is that distribution is not governed solely by plasma protein binding, but by the balance between binding to plasma proteins and to tissue phospholipids. Our biomimetic HPLC studies showed that the unbound volume of distribution is well described by the combined contribution of albumin and IAM binding, supporting the view that both processes reduce the overall free concentration of drug in vivo. Positively charged compounds tend to bind more strongly to phospholipid membranes, whereas negatively charged compounds tend to bind more strongly to albumin, meaning that tissue-to-plasma partition is strongly influenced by molecular charge rather than octanol/water lipophilicity alone [9].

The colour code shows the acid/base character of the compound. Red means that the compound has a significant negative charge at pH 7.4, while the blue colour represents compounds that have a significant amount of positive charge at physiological pHs. The green colours represent compounds that have no charge at pH 7.4, the shade reflects the presence of a weak acidic or weak basic group that can be charged at a higher or lower pH than the physiological pH.

Furthermore, drug efficiency [11], reflecting the balance between dose and free drug exposure, exhibited a clear positive association with the clinical unbound volume of distribution (calculated from the clinical *V*_d_ and the plasma protein binding.

Multivariate analysis, as shown by Equation 7, incorporating albumin binding, phospholipid binding, and half-life, demonstrated that biomimetic binding descriptors contribute significantly to the variability in systemic clearance (*R*^2^ ≈ 0.39, *p* < 0.0001). Both albumin and IAM binding were statistically significant predictors, with opposing effects consistent with their mechanistic roles in plasma and tissue binding, respectively. However, the moderate predictive power of the model highlights that clearance is governed by additional factors, including intrinsic metabolic activity and elimination pathways. These results support the conclusion that non-specific binding contributes to, but does not solely determine, clearance behaviour.



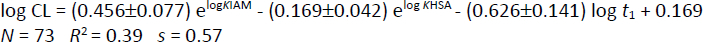

(7)


where N is the number of compounds, and *s* is the standard error of the estimate.

These observations indicate that non-specific binding processes, particularly tissue interactions, play a central role in modulating both distribution and clearance, linking these pharmacokinetic parameters through a common mechanistic framework.

## Conclusions

This work demonstrates that the apparent relationship between volume of distribution and clearance can be mechanistically understood through their shared dependence on non-specific binding processes. While these pharmacokinetic parameters are conceptually independent, both are influenced by the fraction of drug unbound in plasma and tissues.

Analysis of marketed drugs confirms that tissue binding, as captured by phospholipid affinity measured using IAM chromatography, plays a dominant role in determining drug distribution, whereas plasma protein binding alone has limited predictive value when considered in isolation.

The results are consistent with the well-stirred model, where the unbound fraction governs the availability of the drug for elimination. However, clearance remains a multifactorial parameter influenced by intrinsic metabolic activity and elimination pathways, with non-specific binding acting as a modulating factor.

Overall, biomimetic chromatography provides a practical and mechanistically informative approach for integrating binding data into pharmacokinetic models, supporting early estimation of distribution properties and improving understanding of drug disposition in drug discovery.
